# Supplementary material for: A planar neuromuscular controller to simulate compensation strategies in the sit-to-walk movement
Source: PLoS One. 2024 Jun 13;19(6):e0305328. doi: 10.1371/journal.pone.0305328 (PMC11175457; doi:10.1371/journal.pone.0305328)
Supplement: S1 File — File containing data comparing model parameters and muscle moment arms between the H1120 model and commonly used OpenSim models. (PDF) [file pone.0305328.s001.pdf]

Supporting Files

A planar neuromuscular controller to simulate compensation strategies in the sit-to-walk movement.

Table 1 – Comparison of model parameters of the H1120 musculoskeletal model with commonly used OpenSim models: G2392 ((1)) , G1018 ((2)), Rajag (3), Arnold ((4))

		**Max. Isometric Force (N)**					**Optimal Fiber Length (m)**					**Tendon Slack Length (m)**					**Pennation angle (rad)**				
		G2392	*G1018*	*RAJAG*	*ARNOLD*	*H1120*	*G2392*	*G1018*	*RAJAG*	*ARNOLD*	*H1120*	*G2392*	*G1018*	*RAJAG*	*ARNOLD*	*H1120*	*G2392*	*G1018*	*RAJAG*	*ARNOLD*	*H1120*
Biceps Femoris Short head	BFSH	**804**	804	557	705	**804**	0.173	0.173	**0.11**	0.098	**0.11**	0.089	0.089	0.106	0.322	0.1	0.401	0.401	0.264	**0.215**	**0.215**
Gastrocnemius	GAS		2500			2241		0.06			**0.051**		0.39			0.392		0.297			**0.173**
Gastrocnemius lateralis		683		1575	437		0.064		0.059	0.053		0.38		0.376	0.356		0.14		0.21	0.209	
Gastrocnemius medialis		1558		3116	606		0.06		**0.051**	0.059		0.39		0.399	0.382		0.297		0.166	**0.173**	
Gluteus Maximus	GMAX		1944			1944		0.147			**0.147**		0.127			0.127		0			**0**
Gluteus Maximus (superior)		573		984	109		0.142		0.147	0.024		0.125		0.049	0.039		0.087		0.354	0.382	
Gluteus Maximus (middle)		819		1406	546		0.147		**0.157**	0.147		0.127		0.068	0.05		0		0.367	**0.382**	
Gluteus Maximus (inferior)		552		948	781		0.144		0.167	0.157		0.145		0.07	0.073		0.087		0.382	0.382	
Hamstrings	HAM		2700			2594		0.109			**0.098**		0.326			0.325		0			**0.202**
Biceps Femoris Long head		896		1313	324		0.109		**0.098**	0.106		0.326		0.325	0.043		0		0.176	**0.202**	
Semimenbranosus		1288		2201	114		0.08		0.069	0.403		0.359		0.348	0.11		0.262		0.255	0.264	
Semitendinosus		410		591	1163		0.201		0.193	0.069		0.256		0.247	0.348		0.087		0.241	0.225	
Iliopsoas			2342					0.1					0.16					0.14			
Iliacus	ILIAC	1073		1021	137	1073	0.1		**0.107**	0.228	**0.107**	0.1		0.096	0.166	0.093	0.122		**0.28**	0.25	**0.28**
Psoas	PSOAS	1113		1427	296	1113	0.1		**0.117**	0.026	**0.117**	0.16		0.1	0.115	0.197	0.14		**0.216**	0.187	**0.216**
Rectus Femoris	RFEM	1169	1169	2192	254	1169	0.114	0.114	**0.076**	0.054	**0.114**	0.31	0.31	0.448	0.024	0.305	0.087	0.087	0.217	**0.243**	**0.087**
Soleus	SOL	3549	5137	6195	302	3549	0.05	0.05	**0.044**	0.193	**0.044**	0.25	0.25	0.277	0.245	0.243	0.436	0.436	0.381	**0.494**	**0.494**
Tibialis Anterior	TA	905	3000	1227	155	1759	0.098	0.098	**0.068**	0.095	**0.098**	0.223	0.223	0.24	0.45	0.223	0.087	0.087	0.195	**0.168**	**0.087**
Vasti	VAS		5000			4530		0.087			**0.099**		0.136			0.115		0.052			**0.253**
Vastus Intermedius		1365		1697	906		0.087		**0.099**	0.038		0.136		0.202	0.282		0.052		0.063	**0.079**	
Vastus Lateralis		1871		5149	1024		0.084		0.099	0.099		0.157		0.221	0.106		0.087		0.253	0.321	
Vastus Medialis		1294		2748	2255		0.089		0.097	0.099		0.126		0.2	0.13		0.087		0.422	0.517	

MUSCLE MOMENT ARMS

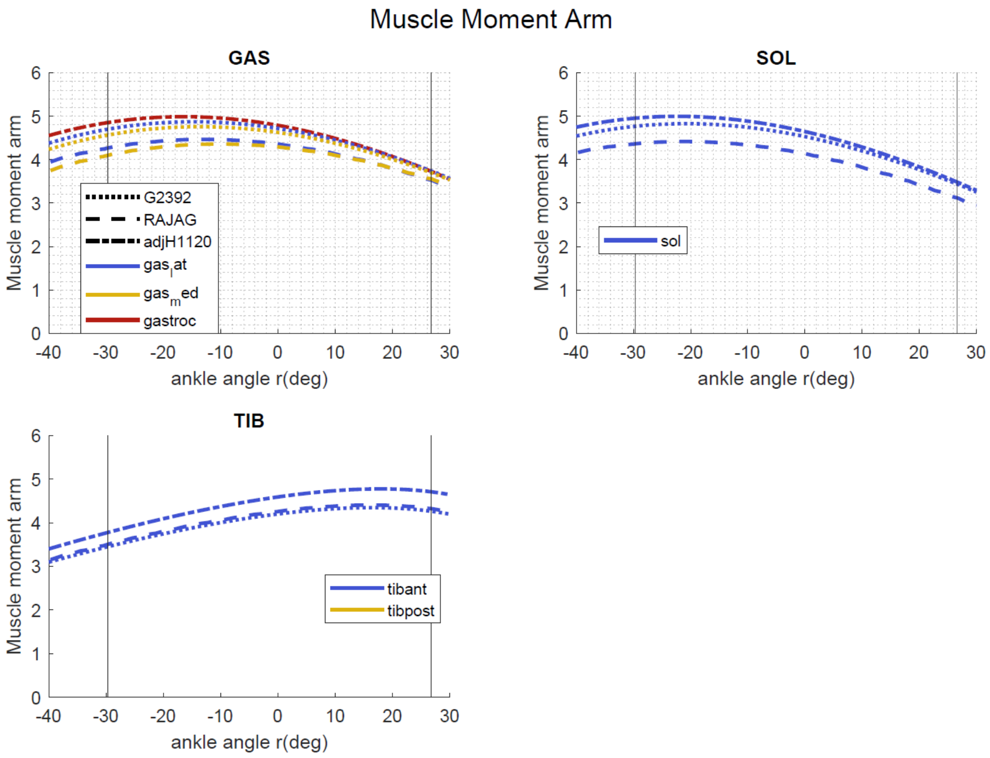

Figure 1 – Muscle Moment arms ankle joint. All other joints are in neutral (extended) positions

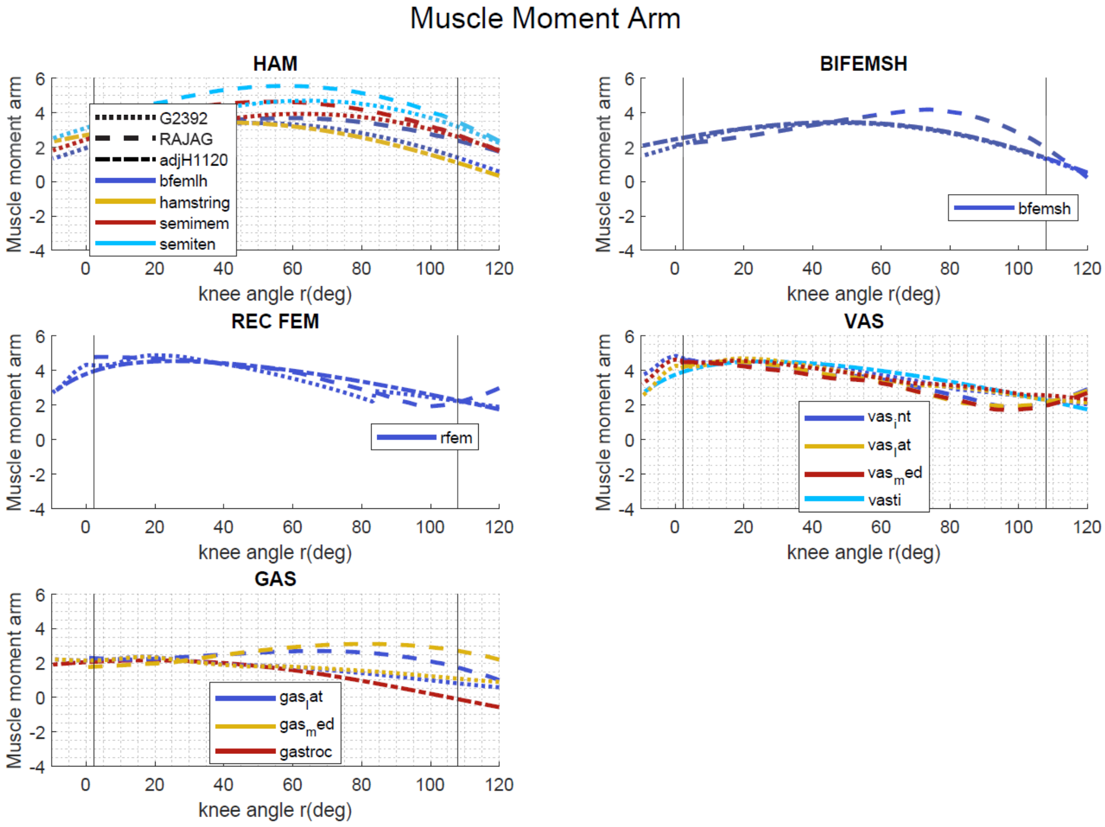

Figure 2 – Muscle Moment arms knee joint. Other joints are in neutral positions.

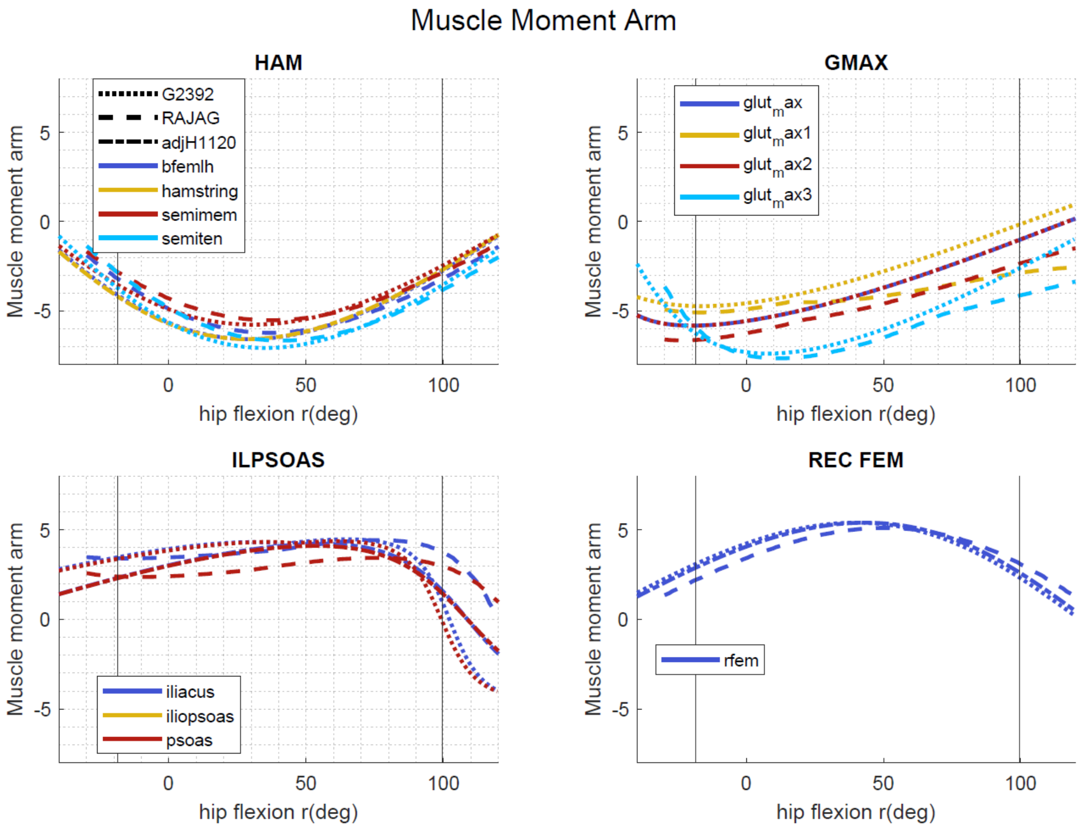

Figure 3 – Muscle Moment arms hip joint. Other joints are in neutral positions.

NORMALIZED FIBER LENGTHS

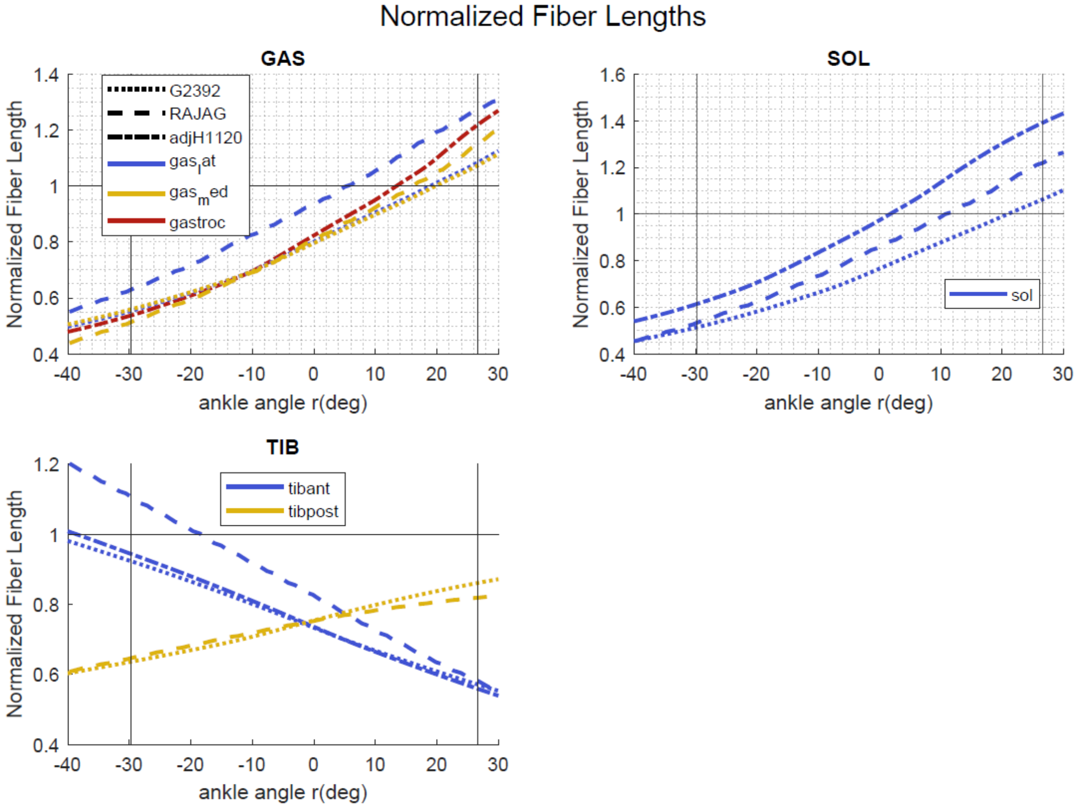

Figure 4 – Normalized Fiber Lengths of the ankle joint. Other joints are in neutral position.

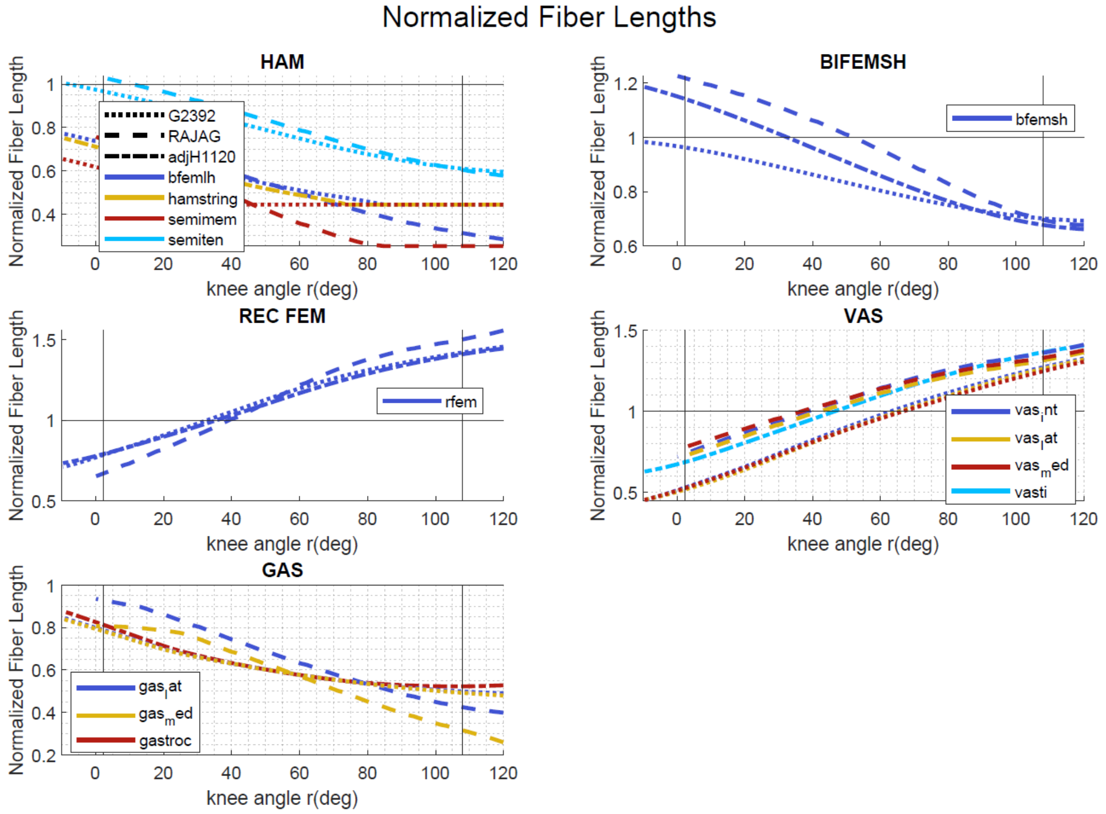

Figure 5 – Normalized Fiber Lengths of the knee joint. Other joints are in neutral position.

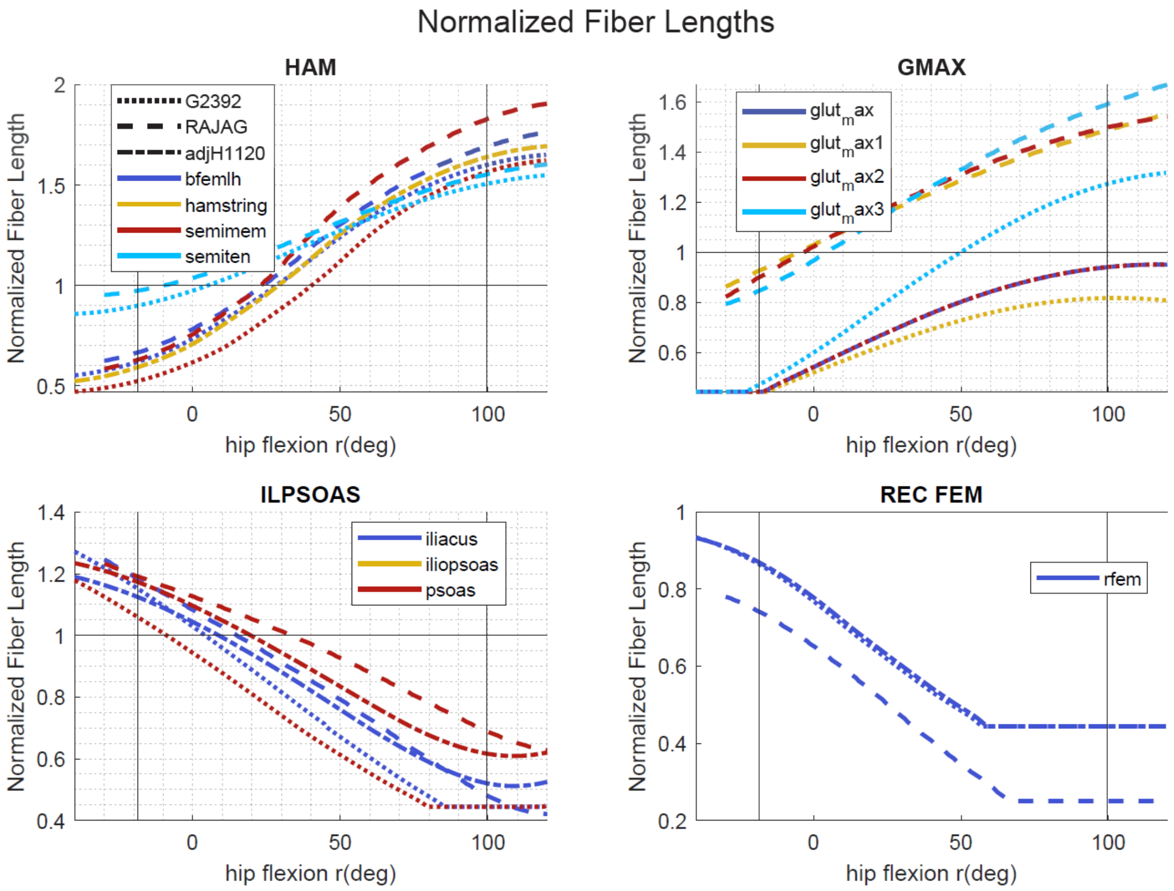

Figure 6 – Normalized Fiber Lengths of the hip joint. Other joints are in neutral position. Normalized fiber length of the Gluteus Maximus follows the G2392 shape. This means that the optimal fiber length for this muscle occurs at a much higher hip flexion (~70deg) than for the RAJAG model (0 deg) (with the knee angle at 0 degrees). The normalized fiber length of the Rectus Femoris flattens at a hip range of >~40 degrees (with the knee angle at 0 degrees). This is a lower than for the RAJAG model (~60 deg) .

NORMALIZED PATHWAY

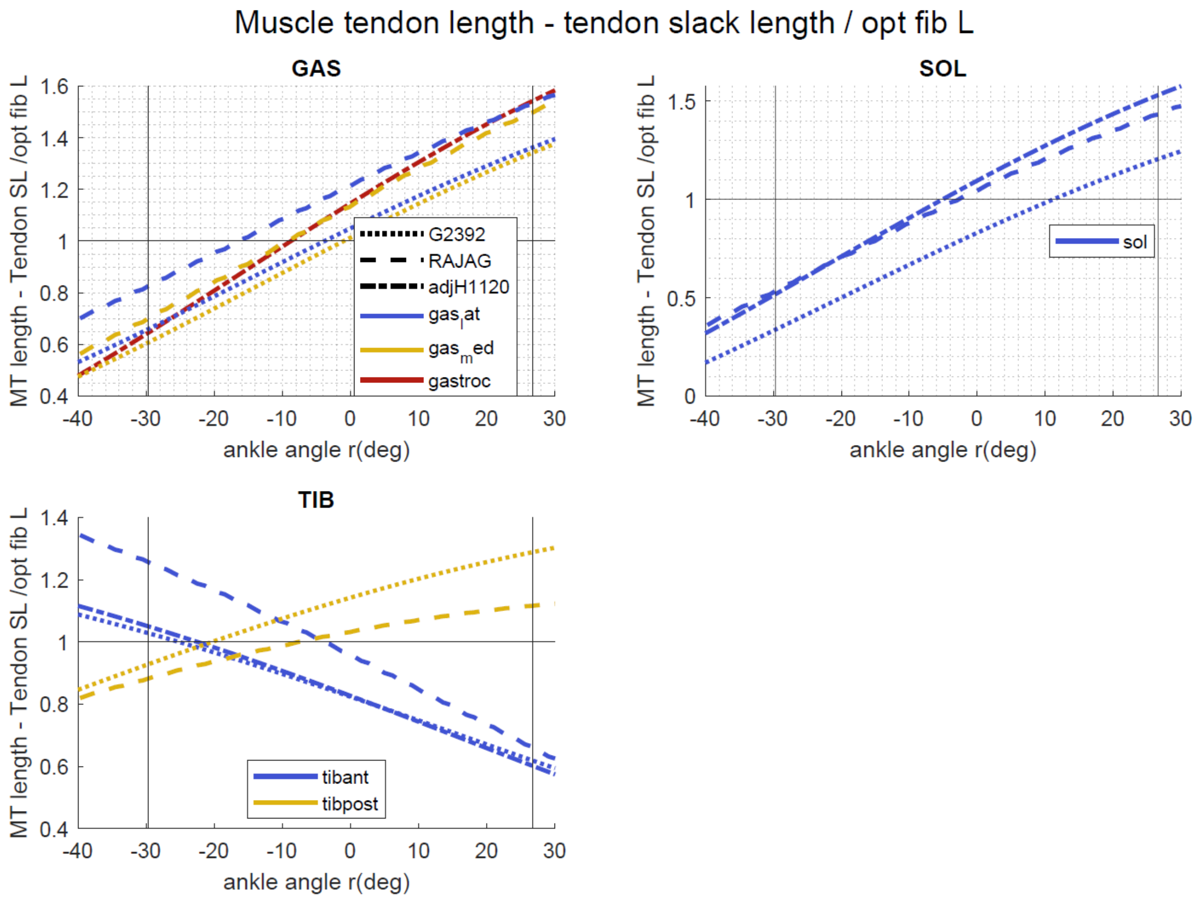

Figure 7 – Normalized Path way of muscle crossing the ankle joint. Other joints are in neutral position. The normalized pathway is estimated by subtracting the tendon slack length from the muscle tendon length and dividing by the optimal fiber length.

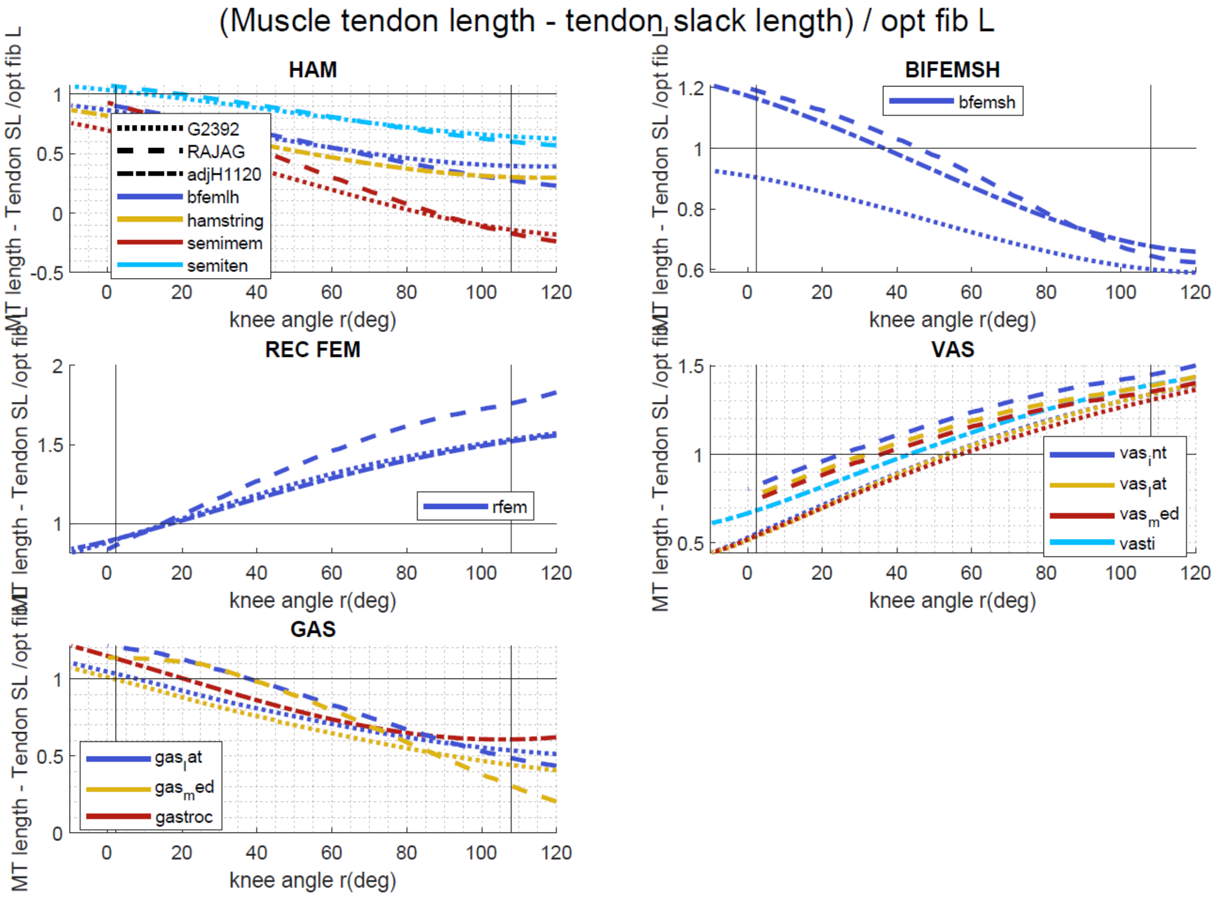

Figure 8 – Normalized Path way of muscle crossing the knee joint. Other joints are in neutral position. The normalized pathway is estimated by subtracting the tendon slack length from the muscle tendon length and dividing by the optimal fiber length. The BFEM follows the G2392 pathway which significantly differs from the RAJAG model. The RFEM follows the G2392 pathway which significantly differs from the RAJAG model.

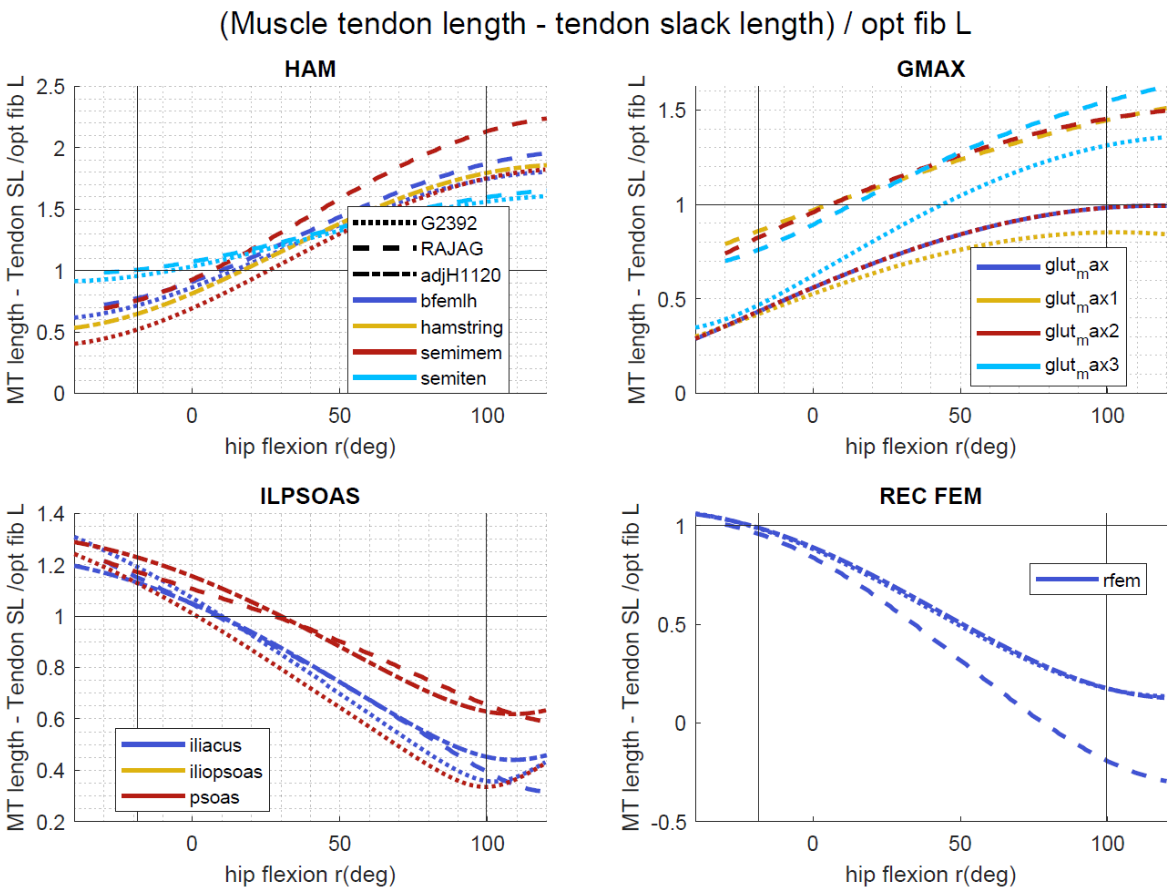

Figure 9 – Normalized Path way of muscle crossing the hip joint. Other joints are in neutral position. The normalized pathway is estimated by subtracting the tendon slack length from the muscle tendon length and dividing by the optimal fiber length.
